# Tracing the Origin, Spread, and Molecular Evolution of Zika Virus in Puerto Rico, 2016–2017

**DOI:** 10.3201/eid2711.211575

**Published:** 2021-11

**Authors:** Gilberto A. Santiago, Chaney C. Kalinich, Fabiola Cruz-López, Glenda L. González, Betzabel Flores, Aaron Hentoff, Keyla N. Charriez, Joseph R. Fauver, Laura E. Adams, Tyler M. Sharp, Allison Black, Trevor Bedford, Esther Ellis, Brett Ellis, Steve H. Waterman, Gabriela Paz-Bailey, Nathan D. Grubaugh, Jorge L. Muñoz-Jordán

**Affiliations:** Centers for Disease Control and Prevention, San Juan, Puerto Rico, USA (G.A. Santiago, F. Cruz-López, G.L. González, B. Flores, K.N. Charriez, L.E. Adams, T.M. Sharp, G. Paz-Bailey, J.L. Muñoz-Jordán);; Yale School of Public Health, New Haven, Connecticut, USA (C.C. Kalinich, A. Hentoff, J.R. Fauver, N.D. Grubaugh);; US Public Health Service, Rockville, Maryland, USA (L.E. Adams, T.M. Sharp);; Fred Hutchinson Cancer Research Center, Seattle, Washington, USA (A. Black, T. Bedford);; US Virgin Islands Department of Health, Charlotte Amalie, St. Thomas, Virgin Islands, USA (E. Ellis, B. Ellis)

**Keywords:** Zika, Zika virus, next-generation sequencing, NGS, Puerto Rico, phylogenetics, genomic epidemiology, viruses, vector-borne infections, molecular evolution, United States

## Abstract

We reconstructed the 2016–2017 Zika virus epidemic in Puerto Rico by using complete genomes to uncover the epidemic’s origin, spread, and evolutionary dynamics. Our study revealed that the epidemic was propelled by multiple introductions that spread across the island, intricate evolutionary patterns, and ≈10 months of cryptic transmission.

Puerto Rico reported the first confirmed case of Zika virus (ZIKV) disease in November 2015 and subsequently experienced epidemic transmission that peaked by mid-August 2016 (*1*). Despite the large number of confirmed cases detected by traditional surveillance, the origin, spread, and evolutionary dynamics of this epidemic remain undetermined. We sought to reconstruct the epidemic transmission period by using a genomic epidemiology approach and determine evolution of the virus in the island.

To investigate the emergence and subsequent epidemic of ZIKV in Puerto Rico, we generated 83 complete genomes (*2*,*3*) directly from PCR-positive serum samples (*4*) (Appendix) collected from the 8 health regions of Puerto Rico during March 2016–January 2017, congruent to a geotemporal representation of the epidemic in the island. We then performed phylogenetic analysis with an additional 233 published genomes from GenBank that represent the emergence and spread of ZIKV in the Americas during 2015–2017. The resulting reconstructed phylogeny was consistent with published tree topologies, nucleotide substitution rate ranges, and divergence patterns observed elsewhere for the entirety of the Americas (Appendix Figure 1, panel A), providing a pragmatic context to the proposed model of spread and divergence of ZIKV in Puerto Rico (*5*). At least 8 separate foreign-introduction events were captured within the ancestry of the viruses sequenced, including 2 that expanded into autochthonous lineages and 6 separate introduction events represented by individual sequences associated with genomes from the United States, the Caribbean, South America, and Central America, thus suggesting limited spread. 

In addition, we analyzed the temporal molecular evolutionary signal in our dataset by reconstructing time-calibrated phylogenies by using genomes annotated with date of sample collection based on year, month, and days for temporal precision. The correlation between date of sample collection and root-to-tip genetic distance supported the heterochronous nature of our dataset. The estimated divergence from the root (i.e., time of most recent common ancestor [tMRCA] of this tree) occurred in February 2013 (because 2013–2014 ZIKV genomes from French Polynesia were used as the root), and the within-epidemic evolutionary rate was 1.09 × 10^−3^ substitutions/site/year (Appendix Figure 1, panel B).

Bayesian reconstruction of Puerto Rico clade 1 (PR C1) presents the largest autochthonous monophyletic cluster that originated from viruses from South America and the Caribbean, including Brazil, Suriname, French Guyana, the US Virgin Islands, and Dominican Republic (Figure). tMRCA estimates place the divergence of PR C1 in mid-June 2015 (95% highest posterior density [HPD] February 2015–October 2015) and a within-outbreak evolutionary rate of 1.61 × 10^−3^ (95% HPD 1.13–2.10 × 10^−3^) substitutions/site/year. In addition, PR C1 was observed to diverge further into 2 subclades (SC1 and SC2) spreading across the island. The second clade, Puerto Rico clade 2 (PR C2), presents a smaller autochthonous monophyletic cluster that originated from viruses in Central America, including Nicaragua and Honduras (Figure). Our tMRCA estimates placed the emergence of PR C2 in February 2016 (95% HPD October 2015–April 2016) and its evolutionary rate was similar to PR C1 at 1.87 × 10^−3^ (95% HPD 1.1–2.64 × 10^−3^). We compared the ZIKV epidemic history of Puerto Rico to the time-calibrated Bayesian phylogenies and observed that the tMRCA of PR C1 precedes the initial confirmation of ZIKV in the island through traditional surveillance methods by 3–10 months and that expansion of all PR lineages coincides with the peak of the epidemic curve (Figure). We assessed phylogenetic clustering patterns for geographic association with each of the health regions and detected none (Appendix Figure 2).

**Figure:**
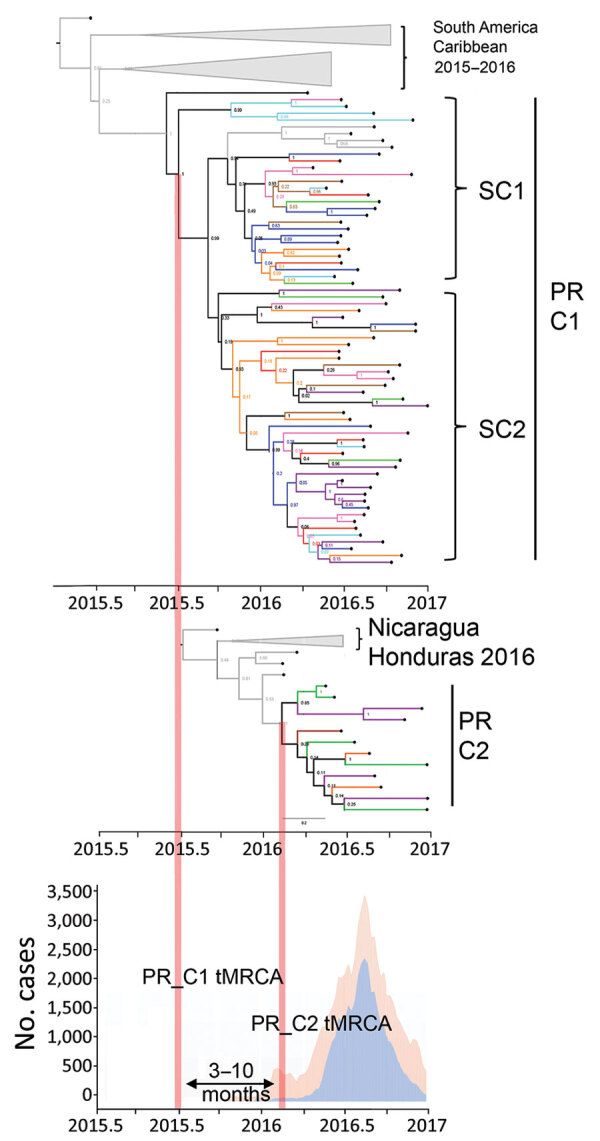
Intra-island spread and divergence of Zika virus, Puerto Rico, 2016–2017. Bayesian phylogenetic reconstruction using maximum clade credibility trees shows genomes grouping with 2 separate clusters. PR C1 is associated with genomes from South America and the Caribbean (top); this clade diverged into SC1 and SC2. PR C2 is associated with genomes from Central America (center). Epidemic curve of total Zika cases per week (orange shade) and cases confirmed by reverse transcription PCR per week (blue shade) during 2015–2017 (bottom). All external branches representing Puerto Rico genomes are color-coded according to the 8 health regions of Puerto Rico: region 1, red; region 2, blue; region 3, orange; region 4, green; region 5, purple; region 6, cyan; region 7, brown; and region 8, magenta. C, clade; PR, Puerto Rico; SC, subclade; tMRCA, time of most recent common ancestor.

We inferred past viral population dynamics by using Bayesian Skygrid plots, which show an increase in genomic diversity that coincides in time with the emergence of ZIKV in the Americas, followed by a series of fluctuations in the effective population size, characteristic of the virus spreading rapidly through the region (Appendix Figure 3). In Puerto Rico, we observed a similar sharp increase upon emergence and subsequent patterns that mirror the trends observed in the Americas.

Our study revealed the origin and epidemic spread of ZIKV in the island after a period of cryptic transmission undetected by traditional surveillance. Similar cryptic transmission was reported in Brazil and Colombia (*6*–*8*), where case detection was hindered by the difficulty to capture asymptomatic or mild cases with clinical manifestations that overlap endemic arboviruses and other laboratory testing limitations particular to ZIKV (*9*). The dataset we generated in our study presents a relevant contribution to the geotemporal sampling of ZIKV genomes from the region, enabling the study the evolutionary and epidemic dynamics in the Americas.

The integration of genomic epidemiology to arbovirus surveillance has proven to be central to the ascertainment of disease epidemiology, uncovering information otherwise concealed by the nature of the disease and limitations of surveillance systems. Fundamentally, integrated proactive genomic surveillance may help us to predict virus emergence and mitigate more effectively their regional or global expansion.

Appendix Additional information about tracing the origin, spread, and molecular evolution of Zika virus, Puerto Rico, 2016–2017.
