# Supplementary material for: Tracing the Origin, Spread, and Molecular Evolution of Zika Virus in Puerto Rico, 2016–2017
Source: Emerg Infect Dis. 2021 Nov;27(11):2971–3. doi: 10.3201/eid2711.211575 (PMC8544999; doi:10.3201/eid2711.211575)
Supplement: Appendix — Additional information about tracing the origin, spread, and molecular evolution of Zika virus, Puerto Rico, 2016–2017. [file 21-1575-Techapp-s1.pdf]

Article DOI: https://doi.org/10.3201/eid2711.211575

Tracing the Origin, Spread, and Molecular Evolution of Zika Virus, Puerto Rico, 2016–2017

Appendix

Methods

Sample Selection

The ZIKV genomes sequenced in this study were obtained from residual clinical serum samples collected through two surveillance systems and tested at the CDC Dengue Branch: the Sentinel Enhanced Dengue Surveillance System (SEDSS) operated by Ponce Health Sciences University (PHSU) and the island-wide arboviral disease surveillance system coordinated by PRDH. All residual samples were handled in accordance with the institutional review boards of CDC protocol # 6731. Serum samples meeting all of the following criteria were selected randomly and independently from each of the eight health regions of Puerto Rico (Figure, panel D): collection within 5 days of symptom onset, ZIKV RNA detected by RT-PCR (*1*) with a cycle threshold (CT) value lower than 32, recorded municipality of residence of the case-patient, and sample collection dates during March 2016 to January 2017. An average of 10 samples were selected for sequencing from each health region.

Next-generation Sequencing

Complete ZIKV genomes were generated directly from clinical serum samples using a modification of PrimalSeq, a targeted method using overlapping PCR amplicon sequencing (*2*,*3*). Viral RNA was extracted from selected archived serum samples using the automated MagNA Pure 96 system (Roche) with the MP96 DNA and Viral RNA Small or Large volume protocol suggested by the manufacturer. All RNA samples were re-tested by RT-PCR (*1*) to confirm eligibility by relative RNA concentration using CT value. First-strand cDNA was synthesized with random hexamers using SuperScript IV reverse transcription (ThermoFisher) and overlapping 400 bp PCR amplicons were generated using 2 separate primer pools (*2*) and Q5®

high-fidelity DNA polymerase (New England Biolabs). Only samples presenting clearly visible bands of target size in DNA gel electrophoresis for both primer pools progressed to DNA library preparation. Qualifying PCR products were purified with magnetic beads (AMPure XP, Beckman Coulter) and concentration reassessed using Qubit 4.0 (ThermoFisher). DNA libraries were prepared using NEBNext Ultra II DNA Library Prep Kit for Illumina (New England Biolabs) reducing all reagents volumes to 25% of the suggested manufacturer protocol and the resulting products were screened for size and quality with the 2100 Bioanalyzer instrument (Agilent Technologies) and for quantity using the Qubit 4 fluorometer (ThermoFisher). Libraries that passed QC were pooled and run in MiSeq (Illumina) using the MiSeq Reagent Kit v3 in 600-cycle protocol.

Sequence Analysis and Generation of Consensus Genomes

The resulting sequence reads were assembled into complete ZIKV genomes using the iVar (*3*) computational package designed for assemblies of amplicon-based sequencing. Briefly, reference-based amplicon alignments were performed using bwa-mem. Primers and low-quality reads were trimmed in the pipeline. Samtools was used for sorting, indexing and variant calling (*3*). Consensus genomes were called by iVar for positions with at least 10x depth and a minimum of 75% frequency of the consensus base. All consensus sequences were inspected manually using MEGA X (https://www.megasoftware.net/) or Geneious Prime (https://www.geneious.com/prime/). A total of 91 samples were sequenced but only 83 genomes assembled with more than 75% coding sequence coverage at 10X depth. Only these 83 complete genomes were considered for the rest of this study. All sequence data obtained for this study was submitted to GenBank: accession numbers MW122373-MW122455.

Phylogenetic Analyses

Our final dataset for phylogenetic and evolutionary dynamics analyses comprised 316 complete genomes including the 83 PR genomes obtained for this study and an additional 233 published genomes, mostly from the Americas. A complete list of all the genome sequences used in this study including GenBank accession numbers can be found in the Supplemental Material (Appendix Table). All multiple sequence alignments were generated with MAFFT (*4*) with subsequent manual editing. All multiple sequence alignments were screened for recombination breakpoints using Recombination Detection Program (RDP4) (*5*) and no evidence of recombination was found. Phylogenetic trees for large datasets were reconstructed with

maximum likelihood (ML) methods including model finding functions or GTR+G pre-selected model options and over 1,000 bootstrap replicates using IQ-TREE v1.6.12 software (*6*). The temporal signal of the ML trees and compatibility with molecular clock phylogenies were assessed with TempEst v1.5.3 (*7*). Evolutionary hypothesis testing and further Bayesian phylogenetic trees to study the PR lineages were reconstructed with maximum clade credibility (MCC) methods using BEAST v1.10.4 (*8*). We estimated nucleotide substitution rates (evolutionary rate), effective population size and MCC trees using Bayesian Markov Chain Monte Carlo (MCMC) approach. For all BEAST analyses, the date of sample collection in the format of yyyy-mm-dd was used to time-calibrate tips and the model was parameterized using the SRD or Yang model of nucleotide substitution, strict or relaxed lognormal molecular clocks, and Bayesian constant coalescent or Skygrid model, depending on dataset. All MCMC were run for sufficient length to ensure stationary parameters with statistical errors reflected in 95% highest probability density ranges including 10% burn-in, with ESS values higher than 200 for each tree prior. The resulting trees were visualized in Figtree v1.4.4 software (http://tree.bio.ed.ac.uk/software/figtree). Additional information and real time tracking of ZIKV molecular evolution globally can be found here: https://nextstrain.org/zika.

References

1. Santiago GA, Vázquez J, Courtney S, Matías KY, Andersen LE, Colón C, et al. Performance of the Trioplex real-time RT-PCR assay for detection of Zika, dengue, and chikungunya viruses. Nat Commun. 2018;9:1391. PubMed https://doi.org/10.1038/s41467-018-03772-1

2. Quick J, Grubaugh ND, Pullan ST, Claro IM, Smith AD, Gangavarapu K, et al. Multiplex PCR method for MinION and Illumina sequencing of Zika and other virus genomes directly from clinical samples. Nat Protoc. 2017;12:1261–76. PubMed https://doi.org/10.1038/nprot.2017.066

3. Grubaugh ND, Gangavarapu K, Quick J, Matteson NL, De Jesus JG, Main BJ, et al. An amplicon-based sequencing framework for accurately measuring intrahost virus diversity using PrimalSeq and iVar. Genome Biol. 2019;20:8. PubMed https://doi.org/10.1186/s13059-018-1618-7

4. Katoh K, Misawa K, Kuma K, Miyata T. MAFFT: a novel method for rapid multiple sequence alignment based on fast Fourier transform. Nucleic Acids Res. 2002;30:3059–66. PubMed https://doi.org/10.1093/nar/gkf436

5. Martin DP, Murrell B, Golden M, Khoosal A, Muhire B. RDP4: Detection and analysis of recombination patterns in virus genomes. Virus Evol. 2015;1:vev003. PubMed https://doi.org/10.1093/ve/vev003

6. Nguyen LT, Schmidt HA, von Haeseler A, Minh BQ. IQ-TREE: a fast and effective stochastic algorithm for estimating maximum-likelihood phylogenies. Mol Biol Evol. 2015;32:268–74. PubMed https://doi.org/10.1093/molbev/msu300

7. Rambaut A, Lam TT, Max Carvalho L, Pybus OG. Exploring the temporal structure of heterochronous sequences using TempEst (formerly Path-O-Gen). Virus Evol. 2016;2:vew007. PubMed https://doi.org/10.1093/ve/vew007

8. Suchard MA, Lemey P, Baele G, Ayres DL, Drummond AJ, Rambaut A. Bayesian phylogenetic and phylodynamic data integration using BEAST 1.10. Virus Evol. 2018;4:vey016. PubMed https://doi.org/10.1093/ve/vey016

Appendix Table. Genomic sequences included in phylogenetic analyses.

GenBank accession no.	Country	Sample collection date
MW122373	Puerto Rico	7/20/2016
MW122374	Puerto Rico	4/27/2016
MW122375	Puerto Rico	7/13/2016
MW122376	Puerto Rico	6/28/2016
MW122377	Puerto Rico	7/30/2016
MW122378	Puerto Rico	7/1/2016
MW122379	Puerto Rico	8/15/2016
MW122380	Puerto Rico	8/26/2016
MW122381	Puerto Rico	10/17/2016
MW122382	Puerto Rico	6/23/2016
MW122383	Puerto Rico	6/25/2016
MW122384	Puerto Rico	9/19/2016
MW122385	Puerto Rico	6/23/2016
MW122386	Puerto Rico	7/17/2016
MW122387	Puerto Rico	7/14/2016
MW122388	Puerto Rico	10/31/2016
MW122389	Puerto Rico	9/7/2016
MW122390	Puerto Rico	10/11/2016
MW122391	Puerto Rico	8/6/2016
MW122392	Puerto Rico	11/7/2016
MW122393	Puerto Rico	6/24/2016
MW122394	Puerto Rico	7/14/2016
MW122395	Puerto Rico	7/23/2016
MW122396	Puerto Rico	8/11/2016
MW122397	Puerto Rico	9/27/2016
MW122398	Puerto Rico	9/19/2016
MW122399	Puerto Rico	11/4/2016
MW122452	Puerto Rico	1/19/2017
MW122400	Puerto Rico	11/10/2016
MW122401	Puerto Rico	9/15/2016
MW122453	Puerto Rico	7/27/2016
MW122402	Puerto Rico	7/1/2016
MW122403	Puerto Rico	6/30/2016
MW122404	Puerto Rico	8/15/2016
MW122405	Puerto Rico	8/19/2016
MW122406	Puerto Rico	8/18/2016
MW122407	Puerto Rico	8/31/2016
MW122408	Puerto Rico	9/27/2016
MW122409	Puerto Rico	10/20/2016

GenBank accession no.	Country	Sample collection date
MW122410	Puerto Rico	10/25/2016
MW122411	Puerto Rico	10/16/2016
MW122412	Puerto Rico	11/3/2016
MW122413	Puerto Rico	12/11/2016
MW122414	Puerto Rico	8/15/2016
MW122415	Puerto Rico	1/20/2017
MW122416	Puerto Rico	6/4/2016
MW122417	Puerto Rico	6/13/2016
MW122418	Puerto Rico	5/26/2016
MW122419	Puerto Rico	7/9/2016
MW122420	Puerto Rico	8/9/2016
MW122421	Puerto Rico	8/17/2016
MW122422	Puerto Rico	9/7/2016
MW122423	Puerto Rico	10/6/2016
MW122424	Puerto Rico	12/2/2016
MW122425	Puerto Rico	6/29/2016
MW122426	Puerto Rico	7/3/2016
MW122427	Puerto Rico	8/23/2016
MW122428	Puerto Rico	9/15/2016
MW122429	Puerto Rico	10/2/2016
MW122430	Puerto Rico	11/3/2016
MW122454	Puerto Rico	6/27/2016
MW122431	Puerto Rico	11/3/2016
MW122432	Puerto Rico	6/28/2016
MW122433	Puerto Rico	7/27/2016
MW122434	Puerto Rico	7/19/2016
MW122435	Puerto Rico	8/17/2016
MW122436	Puerto Rico	10/9/2016
MW122437	Puerto Rico	10/20/2016
MW122438	Puerto Rico	11/21/2016
MW122439	Puerto Rico	11/29/2016
MW122440	Puerto Rico	8/3/2016
MW122441	Puerto Rico	8/23/2016
MW122442	Puerto Rico	8/26/2016
MW122443	Puerto Rico	8/31/2016
MW122444	Puerto Rico	9/10/2016
MW122445	Puerto Rico	12/8/2016
MW122446	Puerto Rico	6/20/2016
MW122447	Puerto Rico	6/27/2016
MW122448	Puerto Rico	10/4/2016
MW122449	Puerto Rico	12/8/2016
MW122450	Puerto Rico	1/10/2017
MW122451	Puerto Rico	1/3/2017
MW122455	Puerto Rico	1/19/2017
KU312312	Suriname	10/2/2015
KU365778	Brazil	2015
KU497555	Brazil	11/30/2015
KU501216	Guatemala	12/1/2015
KU501217	Guatemala	11/1/2015
KU509998	Haiti	12/12/2014
KU527068	Brazil	2015
KU647676	Martinique	12/2015
KU707826	Brazil	7/1/2015
KU729217	Brazil	2015
KU729218	Brazil	2015
KU758877	French Guiana	12/2015
KU820898	Venezuela	2/14/2016
KU870645	Guatemala	2/2/2016
KU922923	Mexico	2/25/2016
KU926309	Brazil	1/14/2016
KU926310	Brazil	1/29/2016
KU937936	Suriname	2/11/2016
KU991811	Brazil	3/6/2016
KX051563	Haiti	2/5/2016
KX051563	United States	2/5/2016
KX056898	Venezuela	2/25/2016
KX101060	Brazil	05/2015
KX156774	Panama	12/18/2015

GenBank accession no.	Country	Sample collection date
KX156775	Panama	12/11/2015
KX156776	Panama	12/18/2015
KX197192	Brazil	2015
KX197205	Brazil	12/1/2015
KX198135	Panama	2016
KX247646	Colombia	2/9/2016
KX262887	Honduras	1/6/2016
KX269878	Haiti	2/1/2016
KX280026	Brazil	2015
KX446950	Mexico	1/1/2016
KX446951	Mexico	1/1/2016
KX447510	French Polynesia	12/2013
KX447511	French Polynesia	01/2014
KX447513	French Polynesia	12/2013
KX548902	Colombia	10/7/2015
KX694534	Honduras	1/6/2015
KX702400	Venezuela	3/25/2016
KX766028	Dominican Republic	6/6/2016
KX811222	Brazil	6/14/2016
KX832731	United States	8/24/2016
KX879603	Ecuador	04/2016
KX879604	Ecuador	04/2016
KX906952	Honduras	4/16/2016
KY014297	Brazil	4/12/2016
KY014300	Dominican Republic	4/20/2016
KY014303	Dominican Republic	4/11/2016
KY014305	Dominican Republic	4/5/2016
KY014306	Honduras	6/10/2016
KY014307	Brazil	3/28/2016
KY014308	Brazil	3/23/2016
KY014310	Honduras	5/9/2016
KY014311	Honduras	5/12/2016
KY014312	Honduras	5/13/2016
KY014314	Dominican Republic	6/14/2016
KY014315	Honduras	6/6/2016
KY014318	Dominican Republic	4/27/2016
KY014319	Honduras	4/30/2016
KY014321	Dominican Republic	4/11/2016
KY014327	Honduras	6/9/2016
KY075932	Martinique	3/22/2016
KY075937	United States	9/9/2016
KY120349	Mexico	3/3/2016
KY120352	Brazil	3/22/2016
KY272991	Brazil	2/12/2016
KY317936	Colombia	1/16/2016
KY317937	Colombia	1/7/2016
KY317938	Colombia	1/10/2016
KY317939	Colombia	1/6/2016
KY317940	Colombia	1/9/2016
KY325464	United States	9/19/2016
KY325465	United States	9/28/2016
KY325471	United States	8/8/2016
KY325472	United States	8/23/2016
KY325476	United States	10/11/2016
KY325479	United States	9/28/2016
KY325482	United States	8/24/2016
KY328289	Honduras	5/15/2016
KY348640	Suriname	1/22/2016
KY441401	Brazil	2/29/2016
KY441402	Brazil	4/5/2016
KY441403	Brazil	1/11/2016
KY558989	Brazil	2/23/2015
KY558990	Brazil	1/15/2016
KY558991	Brazil	1/19/2016
KY558992	Brazil	1/6/2016
KY558994	Brazil	1/18/2016
KY558995	Brazil	5/13/2015
KY558996	Brazil	5/13/2015

GenBank accession no.	Country	Sample collection date
KY558998	Brazil	6/15/2015
KY558999	Brazil	7/10/2016
KY559000	Brazil	8/9/2015
KY559001	Brazil	8/20/2015
KY559002	Brazil	9/9/2015
KY559003	Brazil	8/28/2015
KY559005	Brazil	4/18/2016
KY559015	Brazil	4/24/2016
KY559016	Brazil	4/25/2016
KY559020	Brazil	3/7/2016
KY559022	Brazil	3/13/2016
KY559025	Brazil	1/15/2016
KY559026	Brazil	1/15/2016
KY559027	Brazil	2/16/2016
KY559032	Brazil	01/2016
KY606272	Mexico	8/5/2016
KY606273	Mexico	6/30/2016
KY606274	Mexico	7/7/2016
KY631492	Brazil	1/8/2016
KY631494	Mexico	10/15/2015
KY648934	Mexico	2016
KY693676	Honduras	8/26/2016
KY693677	Honduras	8/26/2016
KY693678	Peru	6/28/2016
KY693679	Peru	7/11/2016
KY693680	Venezuela	10/19/2016
KY765317	Nicaragua	6/29/2016
KY765320	Nicaragua	5/29/2016
KY765321	Nicaragua	7/10/2016
KY765324	Nicaragua	7/7/2016
KY765326	Nicaragua	6/30/2016
KY765327	Nicaragua	4/22/2016
KY785410	Brazil	3/27/2016
KY785413	Dominican Republic	10/6/2016
KY785414	Honduras	5/28/2016
KY785418	Honduras	5/13/2016
KY785419	Jamaica	6/13/2016
KY785420	Dominican Republic	4/18/2016
KY785424	Jamaica	7/10/2016
KY785425	Dominican Republic	4/4/2016
KY785429	Brazil	4/14/2016
KY785430	Jamaica	9/28/2016
KY785431	Honduras	6/13/2016
KY785433	Brazil	4/8/2016
KY785437	Brazil	3/14/2016
KY785441	Dominican Republic	6/13/2016
KY785442	Honduras	6/4/2016
KY785448	Honduras	6/10/2016
KY785450	Brazil	4/12/2016
KY785451	Martinique	3/22/2016
KY785452	Honduras	6/7/2016
KY785453	Dominican Republic	5/10/2016
KY785454	El Salvador	8/5/2016
KY785455	Brazil	4/6/2016
KY785456	Brazil	4/15/2016
KY785466	Colombia	4/28/2016
KY785469	Colombia	4/5/2016
KY785470	Dominican Republic	6/18/2016
KY785473	Dominican Republic	6/15/2016
KY785475	Dominican Republic	4/7/2016
KY785480	Brazil	3/30/2016
KY989971	Colombia	12/2015
MF073357	Brazil	2/1/2016
MF073358	Brazil	6/1/2015
MF073359	Brazil	3/1/2015
MF098764	Dominican Republic	5/11/2016
MF098765	Dominican Republic	5/23/2016
MF098767	Saint Barthélemy	7/25/2016

GenBank accession no.	Country	Sample collection date
MF098770	Mexico	11/9/2016
MF098771	Mexico	1/30/2017
MF159531	Cuba	4/19/2017
MF352141	Brazil	5/13/2015
MF434516	Nicaragua	8/5/2016
MF434517	Nicaragua	8/19/2016
MF434518	Nicaragua	8/26/2016
MF434521	Nicaragua	7/19/2016
MF434522	Nicaragua	8/29/2016
MF438286	Cuba	2/12/2017
MF593625	Guatemala	9/14/2016
MF664436	Dominican Republic	8/25/2016
MF783072	Haiti	6/27/2016
MF783073	Haiti	5/10/2016
MF794971	Ecuador	5/1/2016
MF801378	Guatemala	7/21/2016
MF801381	Honduras	2016
MF801382	Honduras	2016
MF801383	Honduras	6/3/2016
MF801384	Honduras	2/3/2016
MF801385	Honduras	2/12/2016
MF801386	Honduras	2/24/2016
MF801387	Honduras	3/29/2016
MF801389	Honduras	5/10/2016
MF801393	Mexico	4/22/2016
MF801398	Mexico	5/20/2016
MF801399	Mexico	5/20/2016
MF801400	Mexico	5/20/2016
MF801403	Mexico	5/25/2016
MF801407	Mexico	7/5/2016
MF801408	Mexico	7/5/2016
MF801410	Mexico	7/5/2016
MF801411	Mexico	7/5/2016
MF801414	Mexico	7/5/2016
MF801415	Mexico	7/5/2016
MF801419	Mexico	7/1/2016
MF801422	Mexico	7/1/2016
MF801423	Mexico	6/30/2016
MF801426	Nicaragua	7/25/2016
MF988734	Cuba	8/2017
MG494697	Mexico	11/24/2016
MH063259	Cuba	7/26/2017
MH063260	Cuba	8/8/2017
MH063261	Cuba	9/24/2017
MH063262	Cuba	7/17/2017
MH063263	Cuba	8/5/2017
MH063264	Cuba	8/13/2017
MH063265	Cuba	7/17/2017
MH157195	Mexico	8/24/2016
MH157201	Mexico	6/28/2016
MH157207	Mexico	8/29/2016
MH157208	Mexico	8/22/2016
MH157209	Mexico	7/5/2016
MH157214	Mexico	6/24/2016
MH179341	Colombia	12/10/2015
MH513598	Brazil	12/9/2015
MH513599	Brazil	12/11/2015
MH513600	Brazil	12/11/2015
MH544701	Colombia	1/17/2016
MK049245	Colombia	2/29/2016
MK049246	Colombia	3/9/2016
MK049247	Colombia	1/29/2016
MK049248	Colombia	7/27/2016
MK049249	Colombia	11/2/2016
MK049250	Colombia	6/22/2016
MK049251	Colombia	8/30/2016
MK560178	Cuba	10/9/2017
MK713750	Honduras	5/26/2016

GenBank accession no.	Country	Sample collection date
MK165880	U.S. Virgin Islands	10/14/2016
MK165881	U.S. Virgin Islands	10/17/2016
MK165882	U.S. Virgin Islands	9/27/2016
MK165883	U.S. Virgin Islands	9/8/2016
MK165884	U.S. Virgin Islands	7/19/2016

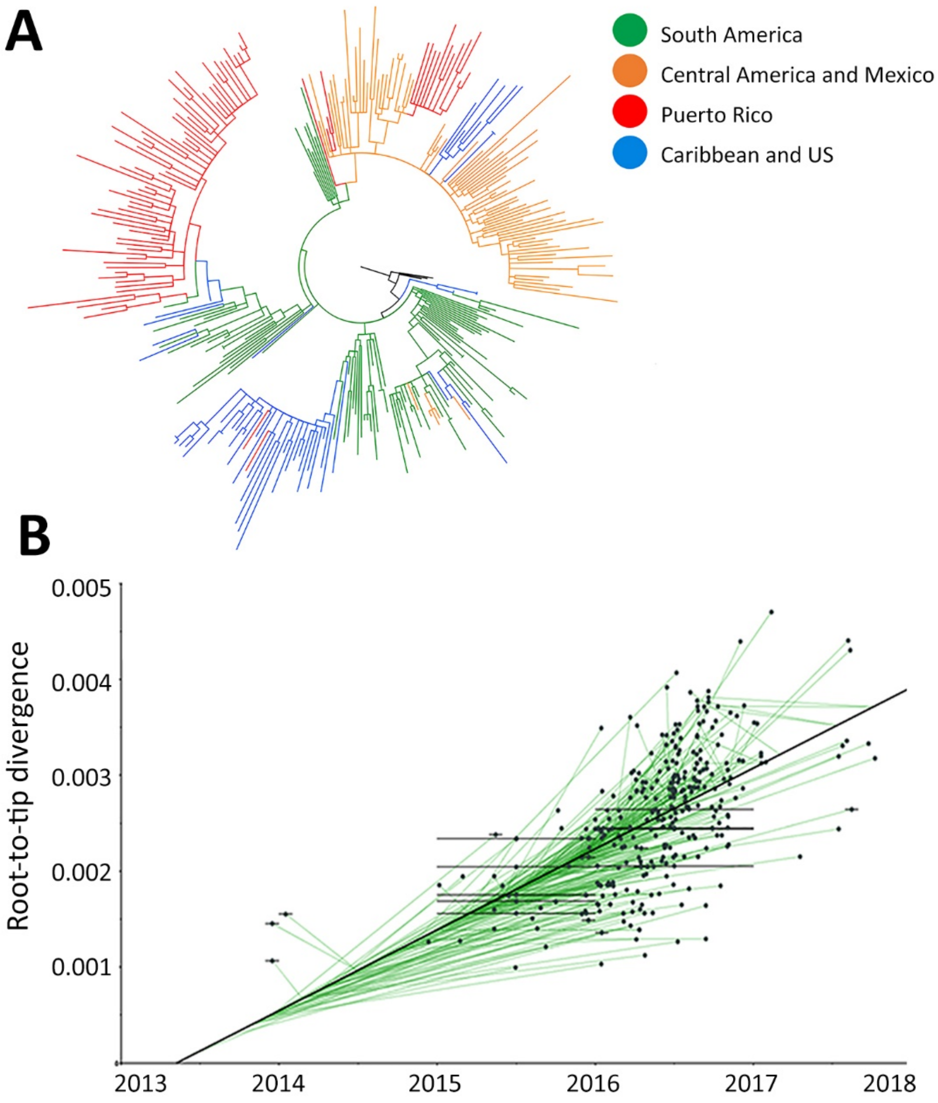

Appendix Figure 1. Emergence and evolution of Zika virus in the Americas and Puerto Rico. (A) Maximum likelihood (ML) phylogenetic inference of 316 complete ZIKV genomes, including 83 genomes from Puerto Rico and 233 genomes from Asia and the Americas obtained from GenBank. The ML tree was rooted to 3 genomes from French Polynesia 2013–2014. Tree is color-coded by geographic region of genome collection; red branches represent PR genomes. (B) Root-to-tip regression analysis showing genome divergence versus sampling time, demonstrating that our dataset follows a molecular clock.

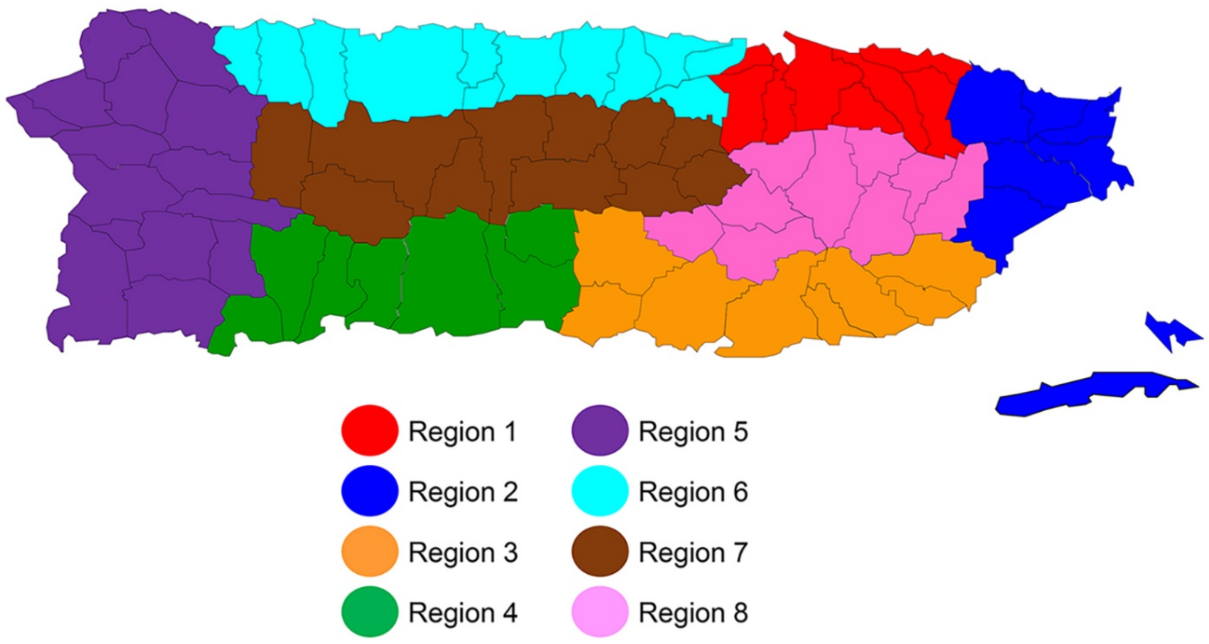

Appendix Figure 2. Map of Puerto Rico health regions. Health regions defined by the Puerto Rico Department of Health.

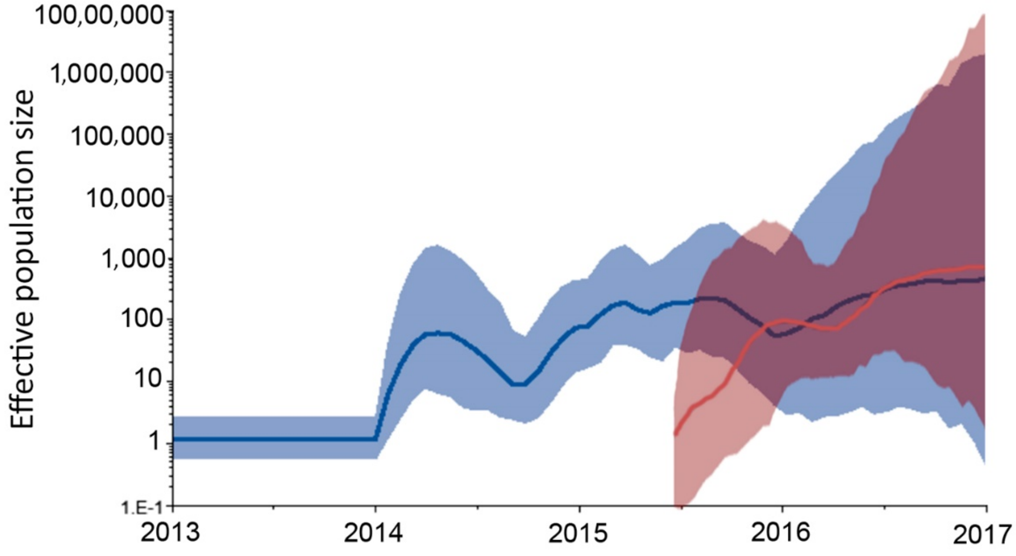

Appendix Figure 3. Genetic diversity of ZIKV in the Americas and in Puerto Rico. Nonparametric estimation of effective population size over time as an inference of past population dynamics from genomic sequence data using Bayesian Skygrid plots. Genetic diversity of ZIKV in the Americas estimated over 150 genomes excluding Puerto Rico (blue plot), compared to the genetic diversity upon emergence in Puerto Rico including 66 genomes from PR clade 1 (red plot).
